# Modifying Effect of the Interleukin-18 Level on the Association between *BDNF* Methylation and Long-Term Cardiovascular Outcomes in Patients with Acute Coronary Syndrome

**DOI:** 10.3390/ijms232315270

**Published:** 2022-12-03

**Authors:** Wonsuk Choi, Hee-Ju Kang, Ju-Wan Kim, Hee Kyung Kim, Ho-Cheol Kang, Sung-Wan Kim, Jung-Chul Kim, Youngkeun Ahn, Myung Ho Jeong, Jae-Min Kim

**Affiliations:** 1 Department of Internal Medicine, Chonnam National University Hwasun Hospital, Chonnam National University Medical School, Hwasuneup 58128, Republic of Korea; 2 Department of Psychiatry, Chonnam National University Medical School, Gwangju 61469, Republic of Korea; 3 Department of Surgery, Chonnam National University Medical School, Gwangju 61469, Republic of Korea; 4 Department of Cardiology, Chonnam National University Medical School, Gwangju 61469, Republic of Korea

**Keywords:** *BDNF* methylation, interleukin-18, acute coronary syndrome, outcome, biomarker

## Abstract

This study investigated the potential modifying effects of the level of the serum interleukin-18 (IL-18) on the association between *BDNF* methylation status and long-term cardiovascular outcomes in patients with acute coronary syndrome (ACS). Hospitalized ACS patients were recruited sequentially from 2006 to 2012. At baseline, the IL-18 level and *BDNF* methylation status were evaluated in 969 patients who were followed for major adverse cardiac events (MACEs) for 5–12 years, until 2017 or death. The time to first composite or individual MACE was compared between individuals with lower and higher average *BDNF* methylation levels (in the low- and high-IL-18 groups, respectively) using a Cox proportional hazards model. After adjusting for potential covariates, the modifying effects of IL-18 and average *BDNF* methylation levels on the initial composite and individual MACEs were examined. In the high-IL-18 group, but not in the low-IL-18 group, a higher average *BDNF* methylation level was associated with increases in composite MACEs (HR (95% CI) = 2.15 (1.42–3.26)), all-cause mortality (HR (95% CI) = 1.89 (1.11–3.22)), myocardial infarction (HR (95% CI) = 1.98 (1.07–3.67)), and percutaneous coronary intervention (HR (95% CI) = 1.81 (1.01–3.23)), independent of confounding variables. The interaction effect between the IL-18 and average *BDNF* methylation levels on composite MACEs (*p* = 0.019) and myocardial infarction (*p* = 0.027) was significant after adjusting for covariates. Analysis of *BDNF* methylation status and IL-18 levels may help identify ACS patients who are most likely to have adverse clinical outcomes.

## 1. Introduction

Neurotrophins contribute to the development of the heart and blood vessels [1,2,3]. The neurotrophin brain-derived neurotrophic factor (BDNF) has attracted much interest as a critical mediator of homeostasis and pathogenesis in the cardiovascular system [4]. A lower CpG methylation level within the regulatory domain of the *Bdnf* gene is related to increased BDNF synthesis in neurons [5], and a higher *BDNF* methylation level has been linked to composite major adverse cardiac events (MACEs) in patients with acute coronary syndrome (ACS) [6].

Inflammation affects the etiology of atherosclerosis and pro-inflammatory cytokines amplify the conventional risk factors for cardiovascular disease (CVD) [7]. Increased inflammatory markers were associated with increased risk of MACEs after adjusting for disease severity or time interval between symptom onset and blood sampling in patients with ACS [8]. Moreover, anti-inflammatory therapy with canalikumab, a therapeutic monoclonal antibody targeting interleukin-1β, led to a significantly lower incidence of recurrent cardiovascular events than placebo in patients with previous myocardial infarction (MI) and a high-sensitivity C-reactive protein level (2 mg or more per liter) [9]. Interleukin-18 (IL-18) [10], a member of the IL-1 cytokine superfamily, is strongly associated with CVD, particularly in ACS patients (who had higher circulating IL-18 levels than controls in a cross-sectional study) [11]. Additionally, higher circulating IL-18 levels were longitudinally associated with a higher risk of death and long-term cardiovascular events in ACS patients [12,13,14]. Furthermore, IL-18 level was an independent predictor of cardiovascular events in subjects with metabolic syndrome [15] or chronic kidney disease [16].

Preclinical studies have shown that pro-inflammatory cytokines inhibit BDNF signaling in the central nervous system (CNS) and affect the process of neurogenesis [17]. Given the substantial association between inflammation and CVD, and the link between inflammation and BDNF signaling in the CNS, pro-inflammatory cytokines may alter the association between BDNF signaling and long-term cardiovascular outcomes. However, this has not yet been investigated.

In this study, we investigated the modifying effect of IL-18 on the association between *BDNF* methylation and long-term cardiovascular outcomes using data from a prospective cohort of ACS patients in Korea. This study is the first prospective investigation to assess the interaction between BDNF and inflammatory pathways regarding long-term cardiovascular outcomes in ACS patients.

## 2. Results

Of the 1152 patients assessed at baseline, 969 (84.1%) agreed to provide blood samples (Figure S1). The baseline characteristics were not significantly different between individuals who consented to provide blood samples and those who did not. Until 2017 or death, all participants were monitored to evaluate cardiovascular outcomes (median; mean (standard deviation) duration of follow-up = 8.4; 8.7 (1.5) years).

The median (interquartile range) and mean (standard deviation) values for IL-18 were 257.4 (126.3) and 271.8 (133.6) pg/mL, respectively. The baseline IL-18 level and *BDNF* methylation were significantly correlated in all 969 study participants (r^2^ = −0.109, *p* = 0.001). Baseline characteristics according to IL-18 level are summarized in Table S1. A high IL-18 level was significantly associated with a higher frequency of hypertension, lower left ventricular ejection fraction (LVEF), and higher frequency of statin use. Based on the data-generation system and potential collinearity between the variables [18], 14 parameters (age, sex, education, Beck Depression Inventory score, previous history of ACS, diabetes, hypertension, hypercholesterolemia, obesity, smoking, LVEF, serum total cholesterol and creatine kinase (CK)-MB levels, and statin use) were included as covariates in the adjusted analysis.

**Figure 1:**
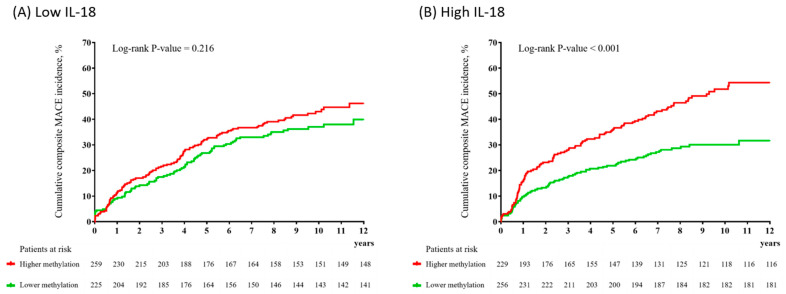
Cumulative incidence (%) of composite major adverse cardiac events (MACEs) according to the average *BDNF* methylation at baseline in patients with low (**A**) and high (**B**) interleukin-18 (IL-18) levels.

In the low-IL-18 group (*n* = 484), the primary outcome (composite MACEs) occurred in 195 participants (40.3%); of the secondary outcomes, all-cause mortality occurred in 91 (18.8%) participants, cardiac death in 51 (10.5%), MI in 43 (8.9), and percutaneous coronary intervention (PCI) in 77 (15.9%). In the high-IL-18 group (*n* = 485), the primary outcome occurred in 188 participants (35.3%), and, of the secondary outcomes, all-cause mortality occurred in 87 (17.9%), cardiac death in 47 (9.7%), MI in 58 (12.0%), and PCI in 62 (12.8%). Figure 1 illustrates the cumulative risk of composite MACEs in subjects with lower versus higher average *BDNF* methylation levels according to the IL-18 level. No significant differences in primary and secondary outcomes were observed in the low-IL-18 group according to methylation level (Figure 1A, Table 1, Figure S2). In the high-IL-18 group, however, a significant difference was observed: the composite MACE incidence was 29.3% (75/256) in those with lower methylation levels and 50.7% (116/229) in those with higher methylation levels (log-rank *p*-value < 0.001) (Figure 1B). In addition, significant differences in those with lower versus higher methylation levels were observed in the incidence of all-cause mortality (13.3% (34/256) vs. 23.1% (53/229), log-rank *p*-value = 0.007), cardiac death (7.0% (18/256) vs. 12.7% (29/229), log-rank *p*-value = 0.037), MI (7.8% (20/256) vs. 16.6% (38/229), log-rank *p*-value = 0.002), and PCI (9.4% (24/256) vs. 16.6% (38/229), log-rank *p*-value = 0.011) (Figure S3). Effects of a higher *BDNF* methylation level on composite MACEs, all-cause mortality, MI, and PCI were also seen in the adjusted analysis (Table 1). In the main analysis, the interaction effect between the IL-18 and average *BDNF* methylation levels on composite MACEs and MI was significant after adjusting for covariates (Table 1). When the average *BDNF* methylation level was treated as a continuous variable, the interaction effect between the IL-18 and average *BDNF* methylation levels on composite MACEs and PCI was significant (Table S2). The IL-18 level had no effect on the incidence of the primary or secondary outcomes in the adjusted analysis (Table S3).

## 3. Discussion

Using data from a prospective study of Korean ACS patients, we showed that the IL-18 level had a modifying effect on the association between *BDNF* methylation and long-term cardiovascular outcomes. In patients with high IL-18 levels, a higher *BDNF* methylation level was a significant predictor of unfavorable long-term cardiovascular outcomes, including composite MACEs, all-cause mortality, MI, and PCI. After adjusting for relevant covariates, the results were still significant. However, among individuals with low IL-18 levels, a higher *BDNF* methylation level had no effect on long-term cardiovascular outcomes.

BDNF signaling pathways and CVD are closely associated. In a cross-sectional investigation, patients with ACS had lower circulating BDNF levels than controls [19]. Additionally, reduced circulating BDNF levels had a deleterious effect on the clinical outcomes of patients with CVD [20,21]. The methylation state of the *BDNF* genomic region that we examined is comparable to a similar area in rat *Bdnf*, which was variably methylated and related to *Bdnf* mRNA expression [22,23]. It has been demonstrated that neurons with elevated BDNF production had lower CpG methylation levels in the regulatory region of the *Bdnf* gene [5]. Previously, we demonstrated that a higher average *BDNF* methylation status was associated with a higher incidence of composite MACEs in ACS patients [6].

Pro-inflammatory cytokines inhibited BDNF signaling pathways in the CNS in preclinical studies [17]. In our study, the IL-18 level and *BDNF* methylation were negatively correlated in the baseline sample. This might be explained by a more severe inflammatory state altering the epigenetics of BDNF signaling. Because both inflammation and BDNF signaling are associated with CVD, and considering the crosstalk between the two pathways in the CNS, pro-inflammatory cytokines might modify the association between BDNF signaling and long-term cardiovascular outcomes. In this study, greater average *BDNF* methylation had a deleterious effect on long-term cardiovascular outcomes only in the high-IL-18 group. The interaction effect of IL-18 and average *BDNF* methylation on composite MACEs was maintained when *BDNF* methylation was examined as a continuous variable. These findings could be attributed to a synergistic effect of both unfavorable exposures (high IL-18 and greater *BDNF* methylation). This notion is supported by the finding that greater *BDNF* methylation had a less deleterious effect in the low-IL-18 group. We further hypothesized that patients with less compensatory epigenetic modification of BDNF signaling in a high inflammatory state would have worse long-term cardiovascular outcomes because the IL-18 level and *BDNF* methylation were negatively correlated in our subjects. However, since we did not assess the causal relationship between IL-18 and *BDNF* methylation, more study is required to confirm this.

Inflammation has a significant impact on the onset and course of CVD [24]. Among pro-inflammatory cytokines, IL-18 has been linked to CVD, especially in ACS. One study reported that ACS patients have higher circulating IL-18 levels than controls [11]. Moreover, other studies reported that higher IL-18 levels predicted worse clinical outcomes in patients with ACS [12,13,14]. In this study, the IL-18 level had no effect on long-term cardiovascular outcomes. Our study was a sub-study of Korean DEPression in ACS (K-DEPACS), which used a naturalistic prospective design to explore the psychological consequences of ACS [25]. In comparison to previous studies, the patients in the current study may have had milder disease, which enabled them to complete the study questionnaire. Since pro-inflammatory cytokine levels and the severity of ACS are correlated [26], our findings on the impact of IL-18 levels on long-term cardiovascular outcomes may have been influenced by the reduced disease severity. The fact that our results differed from those of previous studies may also be due to variation in the time point of blood sampling. In contrast to previous studies that obtained measurements within the first 24 h after admission [12,13,14], we examined the IL-18 levels within 2 weeks of an ACS episode. Since a prior study found that pro-inflammatory cytokine levels in ACS patients peaked at admission and declined thereafter [27], the relatively late assessment of IL-18 levels in our study may have influenced the impact of the IL-18 level on the long-term cardiovascular outcomes.

It is important to consider several limitations when interpreting the findings of this study. First, the *BDNF* methylation status of only one CpG island was evaluated, despite a prior evaluation of this location [22,28]. Second, because of attrition throughout the recruitment process, only 84% of the baseline sample were eligible for methylation analysis. However, there were no differences in baseline clinical or demographic characteristics between patients who had access to this information and those who did not. Third, although the study hypotheses were supported by previous research, mechanistic explanations were lacking, and further investigation is therefore necessary. Finally, the study was limited to a single institution. However, although this reduces its generalizability, it also guaranteed consistency in patient evaluation and care.

This study also had several strengths. It is the first prospective study to examine the interaction between average *BDNF* methylation and IL-18 levels in relation to long-term cardiovascular outcomes in ACS patients. All eligible patients who had recently experienced an ACS episode were enrolled at baseline, limiting the possibility of error due to variation in testing times, and enhancing sample homogeneity. All psychiatric and cardiovascular evaluations were performed using validated measures, and multiple covariates were considered in the analyses.

Our findings imply that both *BDNF* methylation and IL-18 levels should be analyzed to identify patients at risk of recurrence or ACS-related death, and may serve as a predictive biomarker in these patients. Patients with *BDNF* hypermethylation and high IL-18 levels need additional attention from a therapeutic standpoint. Future prospective studies, however, are required to determine whether giving these patients extra care leads to an improved prognosis for ACS patients.

## 4. Methods

### 4.1. Study Overview and Participants

Data from the K-DEPACS study, which uses a naturalistic prospective design to examine the psychological effects of ACS, were analyzed [25]. Figure S4 provides an overview of this study and the participant recruitment strategy. ACS patients who met the eligibility requirements and were hospitalized at the Department of Cardiology of Chonnam National University Hospital in Gwangju, South Korea, were recruited sequentially. For K-DEPACS study enrollment, the inclusion criteria were as follows: (i) age 18–85 years; (ii) ACS confirmed by investigation (the presence of ST-segment elevation MI was defined as >30 min of continuous chest pain, a new ST-segment elevation ≥2 mm on at least two contiguous electrocardiographic leads, and a CK-MB level more than three times the upper limit of normal; the presence of non-ST-segment elevation MI was diagnosed by chest pain and positive cardiac biochemical markers without new ST-segment elevation; and the presence of unstable angina was determined by chest pain within the preceding 72 h with or without ST-T wave changes or positive cardiac biochemical markers); (iii) ability to complete the study questionnaires; and (iv) ability to understand the study objectives and sign informed consent. The exclusion criteria were: (i) occurrence of ACS while hospitalized for another reason; (ii) ACS developing within 3 months after a coronary artery bypass graft procedure; (iii) uncontrolled hypertension (systolic blood pressure > 180 mm Hg or diastolic blood pressure > 100 mm Hg (the same criteria used in the SADHART trial)) [29]; (iv) resting heart rate < 40 beats/min; (v) severe physical illnesses that are life threatening or interfere with ACS recovery; and (vi) persistent clinically significant laboratory abnormalities in complete blood cell counts, thyroid tests, renal function tests, or liver function tests. The research cardiologists treated the ACS of the patients in accordance with international guidelines [30]. Within 2 weeks (mean ± standard deviation: 6.3 ± 2.4 days) following the occurrence of ACS, patients who met the inclusion criteria and consented to take part in the study were evaluated as inpatients for baseline testing. Among these patients, those who consented to blood sampling made up the baseline sample. All participants’ cardiovascular outcomes were monitored until 2017, or until death. This research was authorized by the Institutional Review Board of Chonnam National University Hospital (CNUH I-2008-02-027). All participants provided informed consent.

### 4.2. Primary Measures

#### 4.2.1. Interleukin-18 Level

Participants were instructed to fast the night before blood collection (apart from water). They were told to remain motionless and relax for 25–45 min before the blood samples were taken. The IL-18 solid-phase sandwich Human ELISA kit (Invitrogen, Camarillo, CA, USA) was used to assess the IL-18 level at the Global Clinical Central Lab (Yongin, Korea). A monoclonal antibody specific to IL-18 was coated onto the wells of the microtiter strips provided. 100 μL of samples, standards, and controls were pipetted into the appropriate microtiter wells. During the first incubation, the antigen of each cytokine bound to the immobilized (capture) antibody on one site. After washing, a biotinylated monoclonal antibody specific to each cytokine was added. During the second incubation, this antibody bound to each immobilized cytokine captured during the first incubation. After removal of excess second antibody, streptavidin-peroxidase (enzyme) was added. It bound to the biotinylated antibody to complete the four-member sandwich. After a third incubation and washing to remove all the unbound enzyme, a substrate solution was added, which was acted upon by the bound enzyme to produce color. The intensity of this colored product was measured using a microtiter plate reader (EVOLIS Twin Plus system; Bio-Rad, Hercules, CA, USA) capable of measurement at 450 nm, which was directly proportional to the concentration of each cytokine present in the original specimens. To minimize variations, all samples were analyzed on the same day, in duplicate, and in random order, by a technician blind to the clinical diagnoses. For the initial analysis, patients with low and high IL-18 levels were separated into two groups based on the median value. In subsequent analyses, the IL-18 level was a continuous variable.

#### 4.2.2. BDNF Methylation Status

DNA was extracted from venous blood using conventional methods. The methylation study examined the *BDNF* exon VI promoter region, which has a CpG-rich region with nine CpG sites. Relative to the transcriptional start point in exon VIII, this region is located at nucleotides −612 to −463 (Figure S2). These data have been deposited in GenBank (accession number: BankIt1568919 BDNF JX848620). The *BDNF* region evaluated for methylation was chosen because it corresponds to an analogous region of rat *BDNF* that is differentially methylated and associated with BDNF mRNA expression, and this region has been investigated in the context of antenatal depression [22,31]. Genomic DNA was extracted from leukocytes using the QIAamp DNA Blood Mini Kit (Qiagen, Valencia, CA, USA) following the manufacturer’s suggested protocol. The DNA then underwent bisulfite treatment using the EpiTech Bisulfite Kit (Qiagen) according to the manufacturer’s protocol. A 150-bp fragment of the *BDNF* promoter was amplified by polymerase chain reaction (PCR) from bisulfite-treated DNA using the forward (5′-GTGGGGTAGGAGGGGAGTAGTAT-3′) and reverse (5′-AAATCCCCCAATCAACTCTCT-3′) primers. PCR conditions were 95 °C for 15 min, followed by 45 cycles of 95 °C for 15 s, 57 °C for 30 s, and 72 °C for 15 s, with a final extension of 5 min at 72 °C. PCR products were sequenced using the PSQ 96 M Pyrosequencing System (Biotage, Uppsala, Sweden) according to the manufacturer′s protocol using the sequencing primers (5′-GGTAGGAGGGGAGTAGTA-3′). The methylation percentage at each CpG region was quantified using Pyro Q-CpG software, version 1.0.9 (Biotage). As in prior research [32], the average percentage of *BDNF* methylation was a binary variable (classified as “lower” or “higher”). In subsequent analyses, the average percentage of *BDNF* methylation was analyzed as a continuous variable.

### 4.3. Baseline Covariates

Covariates that potentially affect cardiovascular outcomes were examined within 2 weeks of ACS occurrence. Throughout the evaluation, data on age, sex, years of education, living status (living alone or not), type of residence (owned or rented), and current occupation (employed or not) were collected. Fasting glucose, total cholesterol, blood urea nitrogen, and creatinine levels were assessed using a Hitachi Automatic Analyzer 7600 (Hitachi, Tokyo, Japan). Personal and family histories of depression, as well as the Beck Depression Inventory score [33], were used to evaluate the depression status of the participants. Personal and family histories of ACS, diagnosed diabetes, diagnosed hypertension, hypercholesterolemia based on the fasting serum total cholesterol level (>200 mg/dL) or a history of hyperlipidemia with ongoing treatment, obesity based on measured body mass index (BMI > 25 kg/m^2^), and a reported current smoking status were all used to assess cardiometabolic risk factors. The Killip classification [34] was used to assess current cardiac status, and the LVEF was calculated using echocardiography. Two cardiac enzymes, troponin I and CK-MB, were also examined. Statin use was assessed using presence of a prescription at study enrollment.

### 4.4. Outcomes

As the primary outcome, a MACE was defined as the composite of all-cause mortality, MI, and PCI. Secondary outcomes were all-cause mortality, cardiac death (defined as sudden death for no apparent reason, death from arrhythmias, MI, or heart failure, or death due to heart surgery or endocarditis), MI, and PCI. An independent endpoint committee made up of study cardiologists reached consensus regarding all potential events.

### 4.5. Statistical Analysis

The baseline data were compared according to the IL-18 level (low vs. high) using the independent *t*-test or chi-square test. The covariates used in the adjusted analyses were chosen based on a data-generation system and the variables’ propensity for collinearity [18]. The correlation between the baseline IL-18 level and average *BDNF* methylation level was analyzed using Spearman’s rank-order correlation analysis. The cumulative proportion of participants having composite or individual MACEs (defined by the date of the first incident for each patient) was compared between those with lower and those with higher average *BDNF* methylation levels in the low- and high-IL-18 groups, respectively, using Kaplan–Meier analysis. Cox proportional hazards models were used to compare the time to first composite or individual MACEs, after adjustment for potential covariates, between individuals with lower and those with higher average *BDNF* methylation values in the low- and high-IL-18 groups, respectively. The interaction effect between the IL-18 and average *BDNF* methylation levels on first composite or individual MACEs was analyzed using a Cox proportional hazards model after adjusting for potential covariates. All statistical tests were two-sided, and statistical significance was determined (*p* < 0.05). IBM SPSS Statistics (ver. 25.0) was used for the statistical analysis.

## Figures and Tables

**Table 1:** Associations of higher average *BDNF* methylation at baseline with long-term cardiac outcomes in patients with ACS according to the IL-18 level.

	Low IL-18 (*n* = 484)	High IL-18 (*n* = 485)	*p*-Value for Interaction
Major adverse cardiac events	1.25 (0.83–1.88)	2.15 (1.42–3.26) ^‡^	0.019
All-cause mortality	1.26 (0.76–2.09)	1.89 (1.11–3.22) *	0.192
Cardiac death	1.11 (0.60–2.05)	1.60 (0.81–3.17)	0.250
Myocardial infarction	0.89 (0.47–1.69)	1.98 (1.07–3.67) *	0.027
Percutaneous coronary intervention	1.09 (0.65–1.82)	1.81 (1.01–3.23) *	0.147

HR (95% CI) was adjusted for age, sex, education, Beck Depression Inventory scores, previous history of ACS, diabetes, hypertension, hypercholesterolemia, obesity, smoking, LVEF, serum levels of total cholesterol and creatinine kinase-MB, and statin use at baseline. * *p* < 0.05; **^‡^** *p* < 0.001.

## Data Availability

The data presented in this study are openly available in Genbank at https://www.ncbi.nlm.nih.gov/genbank/ (accessed on 2 December 2022), accession number [BankIt1568919 BDNF JX848620].

## Supplementary Materials

The following supporting information can be downloaded at: https://www.mdpi.com/article/10.3390/ijms232315270/s1.
