# Supplementary material for: Modifying Effect of the Interleukin-18 Level on the Association between BDNF Methylation and Long-Term Cardiovascular Outcomes in Patients with Acute Coronary Syndrome
Source: Int J Mol Sci. 2022 Dec 3;23(23):15270. doi: 10.3390/ijms232315270 (PMC9738340; doi:10.3390/ijms232315270)
Supplement: Supplementary file 1 [file ijms-23-15270-s001.zip › ijms-2021223-supplementary.pdf]

Figure S1. Flow diagram of the recruitment process.

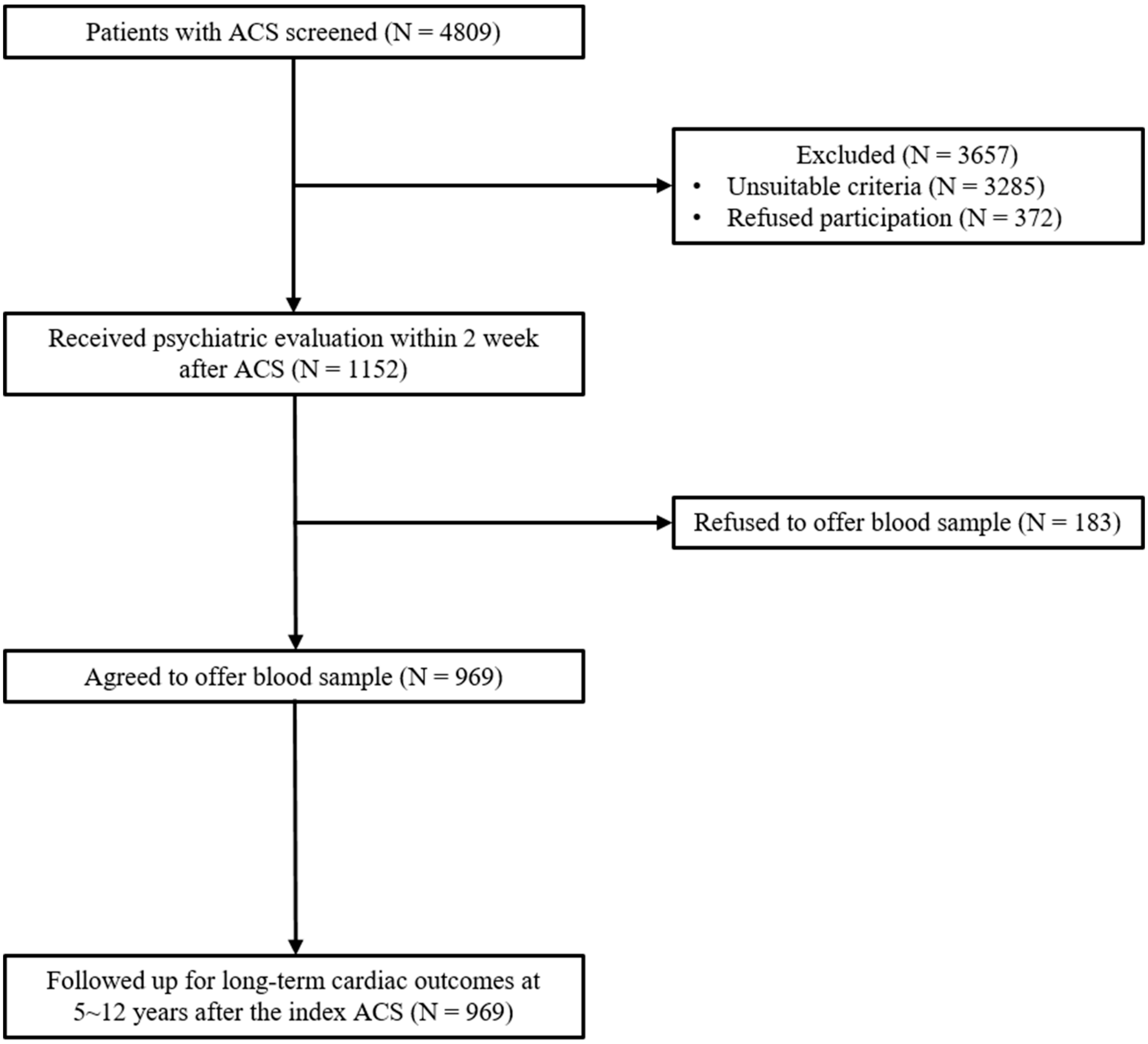

ACS, acute coronary syndrome.

Figure S2. Cumulative incidence (%) of individual major adverse cardiac events according to the average *BDNF* methylation at baseline in patients with low IL-18 levels.

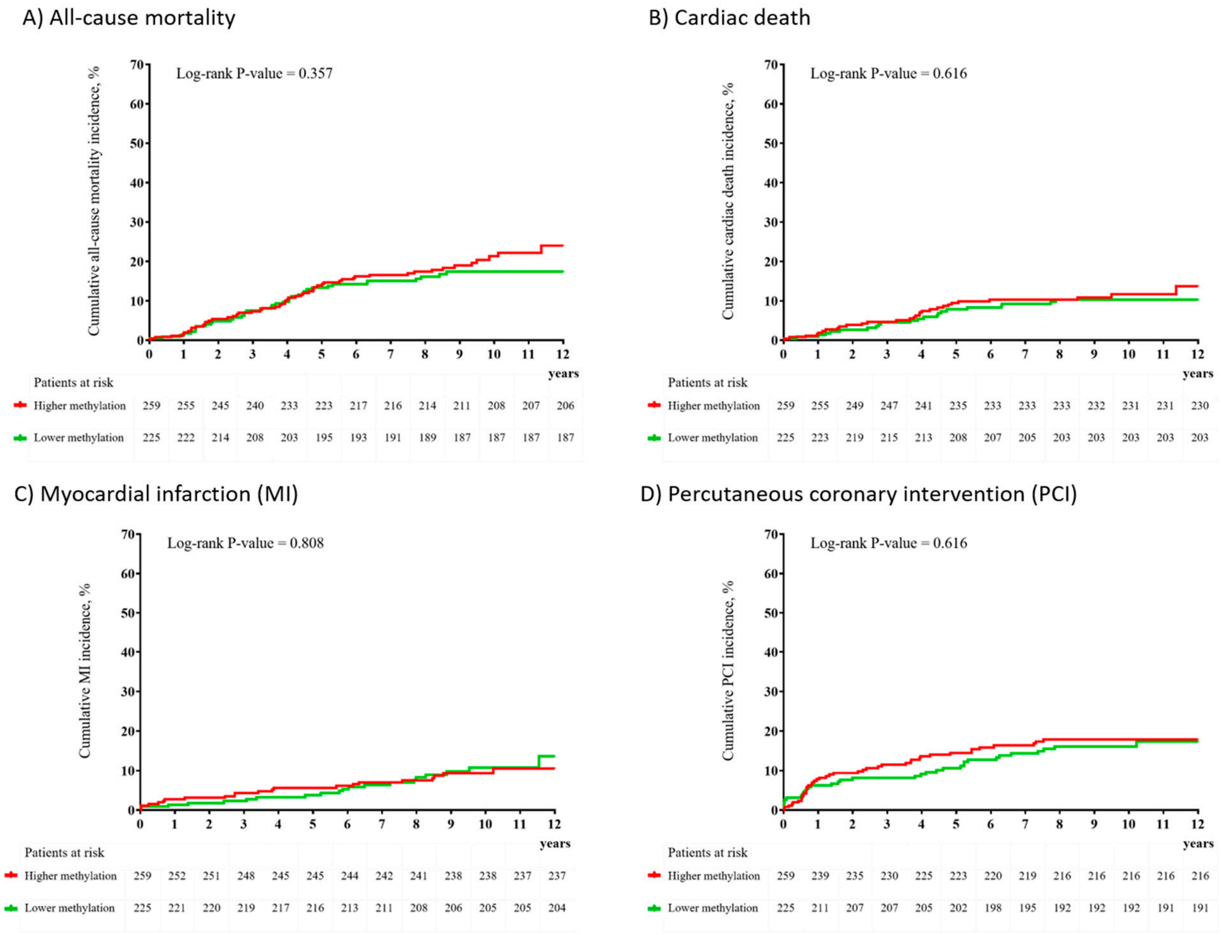

Figure S3. Cumulative incidence (%) of major adverse cardiac events according to the average *BDNF* methylation at baseline in patients with high IL-18 levels.

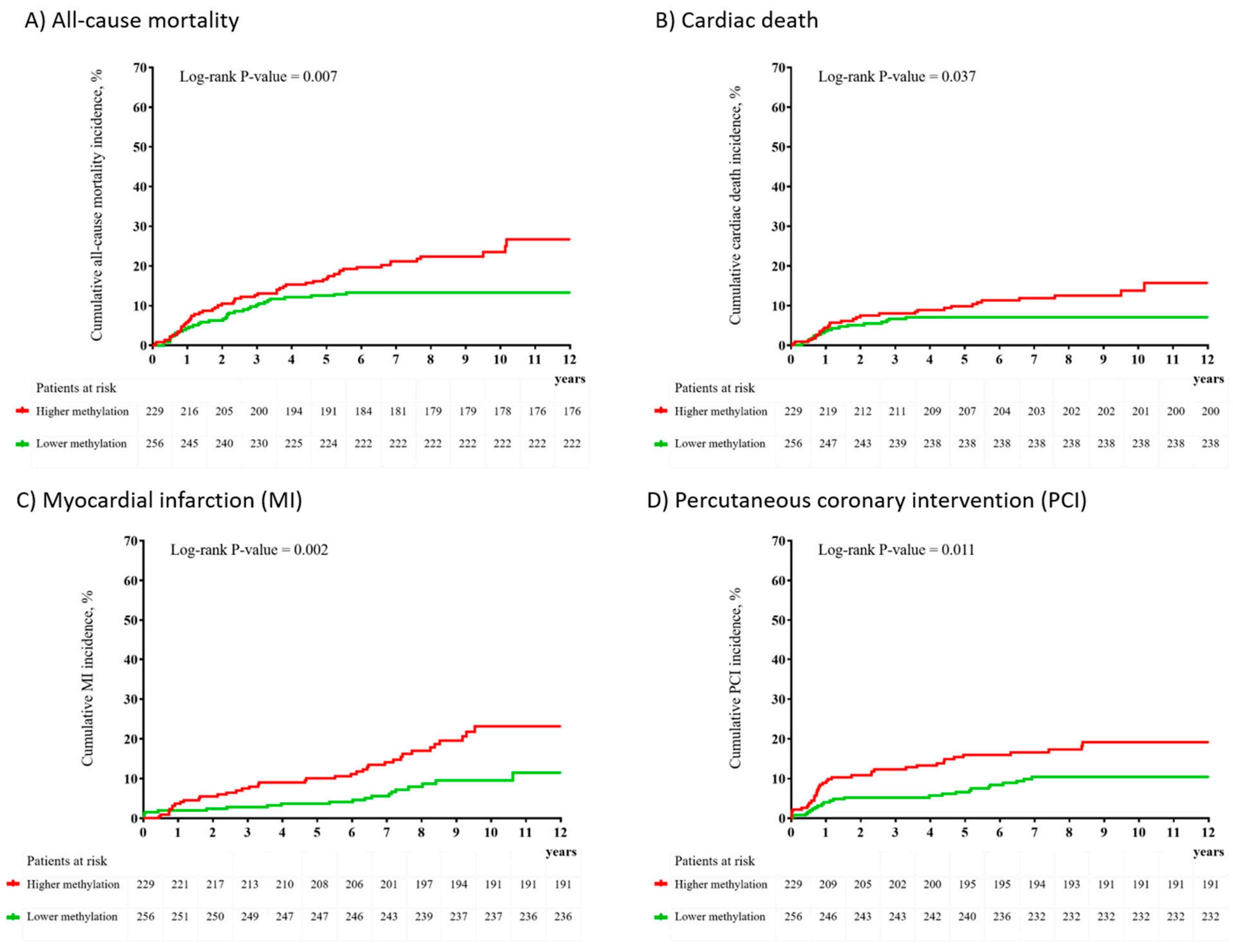

Figure S4. Methylation percentages of CpG regions in *BDNF* exon VI.

Figure legends:

The CpGs are underlined and numbered. Forward and backward primers are shown, as well as sequencers. The genetic sequence is calculated from the transcriptional start site. CpG islands were determined as sequences of at least 200 bp with a GC percentage greater than 50%.

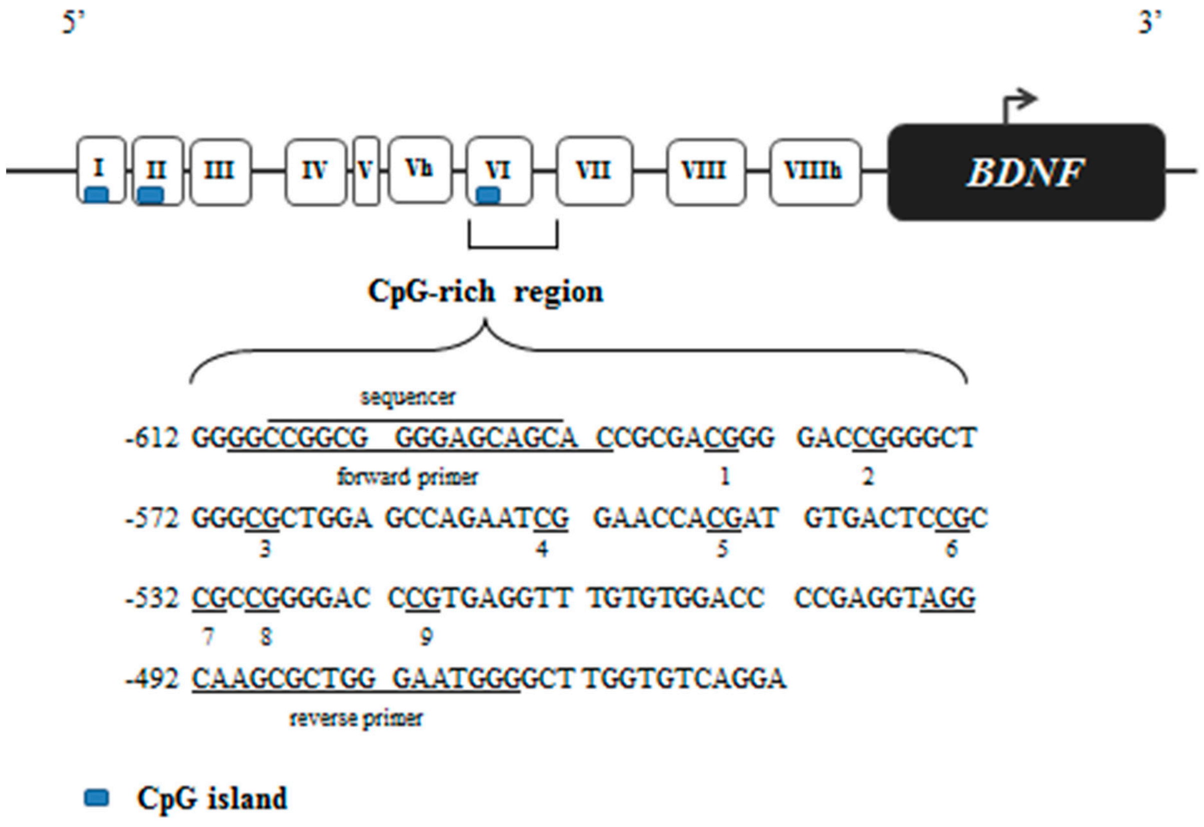

Table S1. Baseline characteristics according to the interleukin-18 (IL-18) level in patients with acute coronary syndrome.

	Low IL-18 (N = 484)	High IL-18 (N = 485)	Statistical coefficient[a]	P-value
**Socio-demographic characteristics**				
Age, mean (SD) years	57.7 (11.1)	58.7 (11.1)	t = -1.457	P = 0.146
Sex, N (%) female	138 (28.5)	131 (27.0)	χ^2 = 0.273	P = 0.616
Education, mean (SD) years	9.9 (4.7)	9.8 (4.7)	t = 0.282	P = 0.778
Marital status, N (%) unmarried	69 (14.3)	72 (14.8)	χ^2 = 0.068	P = 0.795
Living alone, N (%)	48 (9.9)	44 (9.1)	χ^2 = 0.201	P = 0.654
Housing, N (%) rented	66 (13.6)	84 (17.3)	χ^2 = 2.512	P = 0.113
Currently unemployed, N (%)	182 (37.6)	186 (38.4)	χ^2 = 0.057	P = 0.811
**Laboratory assessment**				
Fasting glucose, mean (SD)mg/dL	136.2 (45.5)	134.8 (42.3)	t = 0.510	P = 0.610
Total cholesterol, mean (SD) mg/dL	185.4 (37.0)	185.4 (41.8)	t = 0.011	P = 0.991
BUN, mean (SD) mg/dL	15.4 (11.5)	15.2 (7.5)	t = 0.332	P = 0.740
Creatinine, mean (SD) mg/dL	0.88 (0.29)	0.89 (0.28)	t = -0.490	P = 0.625
**Depression characteristics**				
Previous depression, N (%)	14 (2.9)	20 (4.1)	χ^2 = 1.085	P = 0.298
Family history of depression, N (%)	8 (1.7)	15 (3.1)	χ^2 = 2.167	P = 0.141
BDI, mean (SD) score	9.5 (8.1)	10.5 (9.1)	t = -1.858	P = 0.064
**Cardiac risk factors, N (%)**				
Previous ACS	18 (3.7)	21 (4.3)	χ^2 = 0.234	P = 0.744
Family history of ACS	14 (2.9)	17 (3.5)	χ^2 = 0.294	P = 0.716
Diabetes	85 (17.6)	106 (21.9)	χ^2 = 2.822	P = 0.093
Hypertension	212 (43.8)	246 (50.7)	χ^2 = 4.654	P = 0.031
Hypercholesterolemia	244 (50.4)	242 (49.9)	χ^2 = 0.026	P = 0.872
Obesity	209 (43.2)	206 (42.5)	χ^2 = 0.050	P = 0.824
Current smoker	183 (37.8)	183 (37.7)	χ^2 = 0.001	P = 0.980
**Current cardiac status**				
Killip class >1, N (%)	77 (15.9)	91 (18.8)	χ^2 = 1.377	P = 0.241
LVEF, mean (SD)	62.1 (10.7)	60.2 (11.7)	t = 2.644	P = 0.008
Troponin I, mean (SD) mg/dL	10.2 (16.0)	9.6 (13.8)	t = 0.624	P = 0.533
CK-MB, mean (SD) mg/dL	18.3 (37.0)	16.5 (37.6)	t = 0.731	P = 0.465
Statin use, N (%)	342 (70.7)	370 (76.3%)	χ^2 = 3.937	P = 0.047

[a] Independent two-sample t-test or χ^2 test, as appropriate. BUN, blood urea nitrogen; BDI, Beck Depression Inventory; ACS, acute coronary syndrome; LVEF, left ventricular ejection fraction; CK-MB, creatine kinase-MB.

Table S2. Association of higher average BDNF methylation at baseline with long-term cardiac outcomes in patients with ACS according to the IL-18 level (BDNF methylation is a continuous variable).

	Low IL-18 (N = 484)	High IL-18 (N = 485)	P-value for interaction
Major adverse cardiac events	1.04 (1.00-1.08)	1.07 (1.00-1.13)*	0.033
All-cause mortality	1.06 (1.01-1.12)*	1.07 (1.02-1.13)*	0.798
Cardiac death	1.06 (1.00-1.12)	1.05 (0.97-1.12)	0.868
Myocardial infarction	1.00 (0.95-1.06)	1.08 (1.01-1.15)*	0.065
Percutaneous coronary intervention	0.97 (0.92-1.02)	1.07 (1.00-1.13)*	0.007

HRs (95% CIs) were calculated after adjusting for age, sex, education, Beck Depression Inventory scores, previous history of ACS, diabetes, hypertension, hypercholesterolemia, obesity, smoking, LVEF, serum levels of total cholesterol and creatinine kinase-MB, and statin use at baseline. *$P<0.05$.

Table S3. Effects of the IL-18 level on long-term cardiac outcomes in patients with ACS.

	Major adverse cardiac events	All-cause mortality	Cardiac death	Myocardial infarction	Percutaneous coronary intervention
Low IL-18	Reference	Reference	Reference	Reference	Reference
High IL-18	0.78 (0.59-1.03)	0.80 (0.57-1.14)	0.76 (0.49-1.19)	1.23 (0.80-1.88)	0.71 (0.49-1.03)

Adjusted for age, sex, education, Beck Depression Inventory scores, previous history of ACS, diabetes, hypertension, hypercholesterolemia, obesity, smoking, LVEF, serum levels of total cholesterol and creatinine kinase-MB, and statin use at baseline.

Abbreviations

BDNF, brain-derived neurotrophic factor; MACE, major adverse cardiac event; ACS, acute coronary syndrome; CVD, cardiovascular disease; IL-18, interleukin-18; CNS, central nervous system; LVEF, left ventricular ejection fraction; CK-MB, creatine kinase-MB; PCI, percutaneous coronary intervention; K-DEPACS, Korean DEPression in ACS.
